# Right ventricular dysfunction as predictor of longer hospital stay in patients with acute decompensated heart failure: a prospective study in Indonesian population

**DOI:** 10.1186/s12947-016-0069-0

**Published:** 2016-07-11

**Authors:** Paskariatne Probo Dewi Yamin, Sunu Budhi Raharjo, Vebiona Kartini Prima Putri, Nani Hersunarti

**Affiliations:** 1 Department of Cardiology and Vascular Medicine, Faculty of Medicine, Universitas Indonesia, National Cardiovascular Center Harapan Kita, Jl. Letjend. S. Parman Kav 87, Slipi, Jakarta, 11420 Indonesia; 2 Department of Cardiology, Gatot Subroto Army Hospital, Jakarta, Indonesia

**Keywords:** Acute decompensated heart failure, Right ventricular function, Tricuspid annular plane systolic excursion, Length of stay

## Abstract

**Background:**

Hospital length of stay (LOS) is a key determinant of heart failure hospitalization costs. Longer LOS is associated with lower quality of care measures and higher rates of readmission and mortality. Right ventricular (RV) dysfunction predicted poor outcomes in patients with stable chronic heart failure (CHF), however, its prognostic value in the acute decompensated heart failure (ADHF) patients has not been sufficiently clarified. This study investigated the prognostic value of RV dysfunction in predicting longer LOS in ADHF patients.

**Methods:**

A prospective cohort study was conducted in National Cardiovascular Center Harapan Kita to all patients admitted with ADHF. Clinical data and baseline RV function assessed by tricuspid annular plane systolic excursion (TAPSE) were collected. Clinical comorbidities including malnutrition, pneumonia and worsening renal function (WRF) were monitored during hospitalization. The primary outcome was hospital LOS. Cox regression analysis was used to identify independent predictors for longer LOS.

**Results:**

Two hundred and fifty-nine ADHF patients were included in this cohort study. On time-to-event analysis, diastolic blood pressure (HR = 1.011; 95 % CI = 1.004-1.018; *p* = 0.002), hemoglobin levels (HR = 1.102; 95 % CI = 1.045-1.162; *p* < 0.001), RV function (HR = 0.659; 95 % CI = 0.506-0.857; *p* = 0.002), WRF (HR = 2.015; 95 % CI = 1.520-2.670; *p* < 0.001) and malnutrition (HR = 5.965; 95 % CI = 4.402-8.082; *p* < 0.001) were associated with longer LOS. In a multivariate Cox regression model, RV function (HR = 0.466; 95 % CI = 0.238-0.915; *p* = 0.026), WRF (HR = 2.985; 95 % CI = 2.032-4.386; *p* < 0.001) and malnutrition (HR = 7.479; 95 % CI = 5.071-11.030; *p* < 0.001) were the independent predictors of longer hospital LOS. Based on the median TAPSE values, patients with TAPSE ≤ 16 mm had significantly longer LOS (HR = 2.227; 95 % CI = 1.103-4.494; *p* = 0.026) compared to those with TAPSE > 16 mm.

**Conclusions:**

Right ventricular dysfunction, WRF and malnutrition are important predictors of longer LOS. This is the first study to describe that in ADHF patients, lower the TAPSE resulted in longer the LOS.

## Background

Despite progress in its treatment, heart failure (HF) is a progressive disease that produces high rates of morbidity and mortality, characterized by frequent hospital admissions and prolonged length of hospital stay [1–3]. Acute decompensated heart failure (ADHF), a condition in which the patients experienced rapid worsening of HF signs and symptoms, was the major cause of hospitalization in HF patients [4]. Hospital length of stay (LOS) is a key determinant of HF hospitalization costs. Longer LOS is also associated with lower performance on quality of care measures and higher rates of subsequent readmission and mortality [2, 5]. Therefore accurate prognostic information from determinants associated with increased morbidity, in this case hospital LOS, is required.

Several studies revealed that possible determinants of longer LOS for HF patients include socio-demographic variables, medical comorbidity, disease severity (worse functional class and lower left ventricular ejection fraction), clinical presentation, in-patient treatment, in-hospital progress and the development of iatrogenic complications [1, 6–8]. Some medical comorbidities that were known in prolonging LOS are concurrent stroke, worsening renal function (WRF), atrial fibrillation, respiratory problems requiring specific treatment, and malnutrition [1, 8–11].

In the last few years, attention for right ventricular (RV) structure and function in HF patients has rapidly grown. Although less well studied than the left ventricle (LV), the RV may be of equal importance in determining prognosis in HF patients [12]. RV dysfunction has been shown to be associated with an adverse outcome in patients with symptomatic HF [13–15]. While RV dysfunction has been extensively studied in stable chronic HF, its prognostic value in the setting of ADHF patients has not been sufficiently clarified. Several studies have been conducted with contradictory results [16–18]. Accordingly, this study sought to investigate the prognostic value of RV dysfunction as assessed by TAPSE, and other echocardiographic and clinical comorbidities in predicting longer LOS in ADHF patients.

## Methods

### Patients

This was an observational, prospective cohort study that included ADHF patients aged 18 and above that were hospitalized consecutively in National Cardiovascular Center Harapan Kita (NCCHK) between December 2014 and February 2015. The study was approved by our hospital’s ethical committee.

Diagnosis of ADHF was established according to the recommendations of the European Society of Cardiology (ESC) [19]. Patients were excluded if they had an evidence of acute coronary syndrome, primary valvular heart disease, congenital heart disease, heart failure post valvular and congenital heart surgery, cardiac tamponade, aortic dissection, acute pulmonary embolism, and did not consent to participate in this study. We also excluded patients with severe tricuspid regurgitation on echocardiography examination. Patients who died during hospitalization were also excluded from the study.

### Measurements and study procedures

Baseline and clinical characteristics, anthropometry status, and laboratory data were collected during hospital admission. Clinical comorbidities such as worsening renal function (WRF) and respiratory infection (pneumonia) were monitored during patients’ hospitalization. WRF is the occurrence, at any time during the hospitalization, of ≥ 0.3 mg/dl or ≥ 25 % increase in serum creatinine from admission [20]. Estimated glomerular filtration rate (eGFR) was calculated by the Modification of Diet in Renal Disease (MDRD) equation that has been shown to be the best method for the indirect assessment of renal function in HF patients [21].

### Nutritional assessment

For evaluation of malnutrition, nutritional status was assessed using Nutritional Risk Index (NRI) formula as follows: [1.519 × serum albumin(gr/L) + 41.7(current weight/ideal weight)] [22, 23]. Ideal body weight was calculated using the Lorentz formula [24] for men = [height(cm) ‐ 150]/4] and women = [height(cm) ‐ 150]/2]. Ideal body weight was used instead of usual body weight because it was less subjective. Subjects were classified as having malnutrition if their NRI score was less than 97.5 [25, 26].

### Echocardiographic measurements

Echocardiography examination was performed in the ward within the first 48 h after admission, depends on patient’s clinical condition. It was performed in accordance with the recommendations of the American Society of Echocardiography [27]. LVEF was calculated from apical two- and four-chamber views using the modified Simpson’s rule. RV systolic function was evaluated by M-mode echocardiography using TAPSE. TAPSE was measured on M-mode tracing at the junction of the tricuspid valve and RV free wall in the apical four-chamber view [13]. Data were averaged over 3 beats (5 beats in cases of atrial fibrillation).

### Study outcomes and definitions

The primary outcome in this study is hospital length of stay. Hospital LOS was defined as the actual number of days the patients remained in the hospital, determined from the day of admission to the moment of discharge.

### Statistical analysis

Statistical analysis was performed using SPSS program. Continuous variables are presented as the mean ± standard deviation, or median (minimum-maximum) if not normally distributed. Categorical variables are presented in frequency (percentages). Univariate analysis was performed to identify the variables associated with LOS, including age, clinical characteristics (congestion signs, ischemic etiology, history of hypertension, history of prediabetes/diabetes, atrial fibrillation, systolic blood pressure, diastolic blood pressure, heart rate), laboratory values (hemoglobin, baseline eGFR, random blood glucose), echocardiographic measurements (LV End Diastolic Diameter, LV End Systolic Diameter, LV Ejection Fraction, TAPSE, Mean Pulmonary Artery Pressure), and comorbidities during hospitalization (pneumonia, malnutrition, worsening renal function). Those variables with *p* < 0.25 at the univariate analysis were included in the multivariate model. Backward stepwise selection was then performed to identify independent prognostic factors for hospital LOS. A p value of < 0.05 was considered to be statistically significant. Results are presented as hazard ratios (HR) with confidence interval of 95 %.

## Results

### Patients characteristics

Of a total of 265 ADHF patients that were initially included in this prospective cohort study, 6 patients died during hospitalization period. The 259 study patients had a median age of 59 years old, 74.9 % were male, and 74.1 % had LVEF less than 40 %. Sixty-six percent of patients had prior hypertension, 60.6 % prediabetes/diabetes, and 49 % had a risk factor of smoking. Acute lung edema was diagnosed in 19 (7.3 %) patients and ischemic etiology as the cause of heart failure was discovered in 198 (76.5 %) patients. Forty-nine percent of patients were classified as having malnutrition based on NRI value obtained at hospital admission. There were 31 (12 %) patients that experienced concomitant acute respiratory infections (pneumonia) requiring specific antibiotic therapy and 72 (27.8 %) developed worsening renal function during the course of hospitalization.

During hospital admission, almost all of the patients received intravenous loop diuretics (91.9 %), and an angiotensin converting enzyme inhibitors (ACEi) (56.4 %) or angiotensin receptor blockers (ARB) (35.9 %). The prescription rates of beta blockers and mineralocorticoid receptor antagonist (MRA) were 46.3 % and 49 % respectively. Whereas the usage of calcium channel blockers (CCB) and digoxin on admission were very low, only 7.3 % and 18.1 % respectively.

The median hemoglobin, leucocyte, random blood glucose and albumin were within normal range for our laboratory value. However the median of ureum and creatinine were higher than the normal range. The median of TAPSE and mean pulmonary artery pressure (mPAP) for the entire cohort was 16 mm and 25 mmHg respectively. If we divided TAPSE based on median value of 16 mm, there were 139 (53.7 %) patients with TAPSE ≤ 16 mm and 120 (46.3 %) patients with TAPSE > 16 mm.

Baseline demographic, clinical characteristics, medications on admission, laboratory values and echocardiographic parameters are presented in Table 1.

**Table 1:** Baseline Characteristic of Patients

Variable (N = 259)	Mean ± SD/ Median (min - max Number (%)
Age (year)	59 (18 - 84)
Sex, n (%)	
Male	194 (74.9 %)
Female	65 (25.1 %)
Body mass index (kg/m^2^)	23.87 (12.49 - 49.17)
Nutritional Risk Index (NRI)	98.63 ± 12.37
Risk Factor, n (%)	
Hypertension	171 (66 %)
DM/Prediabetes	157 (60.6 %)
Smoking	127 (49 %)
Clinical Presentation, n (%)	
Acute lung edema	19 (7.3 %)
Increased JVP	194 (74.9 %)
Peripheral edema	154 (59.5 %)
Comorbidities, n (%)	
Pneumonia	31 (12 %)
Worsening Renal Function	72 (27.8 %)
Malnutrition	127 (49 %)
Heart Failure Etiology, n (%)	
Ischemic heart disease	198 (76.5 %)
Hypertensive heart disease	42 (16.2 %)
Other cardiomyopathy	19 (7.3 %)
ECG, n (%)	
Sinus rhythm	213 (82.2 %)
Atrial fibrillation	41 (15.8 %)
Others	5 (1.9 %)
Medications on Admission, n (%)	
Diuretics (Furosemide)	238 (91.9 %)
ACE inhibitors	146 (56.4 %)
Angiotensin receptor blockers (ARB)	93 (35.9 %)
Mineralocorticoid receptor antagonist (MRA)	127 (49 %)
Calcium channel blockers	19 (7.3 %)
Beta blockers	120 (46.3 %)
Digoxin	47 (18.1 %)
Systolic BP (mmHg)	127 (75 - 231)
Diastolic BP (mmHg)	78 (38 - 159)
Heart rate (x/minute)	96 (36 - 179)
Laboratory values	
Hemoglobin (mg/dl)	13.3 (5.8 - 18.8)
Leukocyte (/uL)	8780 (3390 - 29740)
Random blood glucose (mg/dl)	135 (61 - 618)
Albumin (g/dl)	3.5 (1.8 - 4.5)
Ureum (mg/dl)	50 (9 - 278)
Creatinine (mg/dl)	1.4 (0.61 - 10.02)
eGFR	50 (4 - 112)
Echocardiographic parameters	
End Diastolic Diameter (mm)	60 (25 - 92)
End Systolic Diameter (mm)	52 (16 - 83)
Ejection fraction (%)	27 (12 - 85)
≥ 50 %	40 (15.4 %)
40 - 49 %	27 (10.4 %)
< 40 %	192 (74.1 %)
TAPSE (mm)	16 (7 - 29)
> 16 mm	120 (46.3 %)
≤ 16 mm	139 (53.7 %)
Mean Pulmonary Artery Pressure (mmHg)	25 (5 - 50)

### Hospital length of stay

The median length of stay was 7 days, with the shortest hospital stay was 3 days and the longest was 45 days. There were 149 (57.5 %) patients with LOS of ≤ 7 days, and 110 (42.5 %) patients with LOS of > 7 days.

### Predictors of length of stay

From univariate analysis, a longer LOS was found to be associated with higher diastolic BP (HR 1.011; 95 % CI 1.004-1.018; *p* = 0.002), higher hemoglobin level (HR 1.102; 95 % CI 1.045-1.162; *p* < 0.001), lower TAPSE (HR 0.659; 95 % CI 0.506-0.857; *p* = 0.002), worsening renal function (HR 2.015; 95 % CI 1.520-2.670; *p* < 0.001) and malnutrition (HR 5.965; 95 % CI 4.402-8.082; *p* < 0.001).

Multivariate Cox regression analysis was performed in order to investigate independent predictors of longer LOS. Nine variables with *p* < 0.25 from univariate analysis were included in multivariate model (Table 2). In a multivariate Cox regression model, RV function (HR 0.466; 95 % CI 0.238-0.915; *p* = 0.026), WRF (HR 2.985; 95 % CI 2.032-4.386; *p* < 0.001) and malnutrition (HR 7.479; 95 % CI 5.071-11.030; *p* < 0.001) were the independent predictors of longer hospital LOS. When we divided the TAPSE based on its median value, then TAPSE ≤ 16 mm remains significantly associated with longer LOS (HR 2.227; 95 % CI 1.103-4.494; *p* = 0.026).

**Table 2:** Multivariate analysis of length of stay

Variable	HR (95 % CI)	*p*
Congestion signs	1.259 (0.848 - 1.868)	0.253
Prior HT	1.485 (1.019 - 2.162)	0.039
Systolic BP	1.000 (0.990 - 1.009)	0.927
Diastolic BP	1.006 (0.994 - 1.019)	0.333
Hemoglobin	1.041 (0.970 - 1.117)	0.261
TAPSE	0.466 (0.238 - 0.915)	0.026
mPAP	1.003 (0.978 - 1.028)	0.841
Worsening renal function	2.985 (2.032 - 4.386)	*p*<0.001
Malnutrition	7.479 (5.071 - 11.030)	*p*< 0.001

## Discussion

The principal findings in this study are: 1) RV dysfunction as assessed by TAPSE is an independent predictor of longer LOS, and 2) worsening renal function and malnutrition are also associated significantly with longer LOS.

Hospital LOS is increasingly used as a measure of quality care in patients admitted for acute heart failure. Longer LOS is an important outcome, both from the perspective of the patients’ experience and healthcare system [28]. The optimal duration of hospital stay for treatment of an episode of decompensated heart failure remains unknown [1]. The median LOS in our study was 7 days, three days longer than in Acute Decompensated Heart Failure National Registry (ADHERE) [29], but shorter than that reported in EuroHeart Failure Survey II (EHFSII) [30] and Acute Heart Failure Acute Heart Failure Global Survey of Standard Treatment (ALARM-HF) [31].

To the best of our knowledge, our study is the first to describe that RV dysfunction is an independent predictor of longer LOS in the setting of ADHF patients. Higher TAPSE value was found to be a protective factor for longer LOS from multivariate analysis, with HR 0.466 (95 % CI 0.238-0.915; *p* = 0.026). Further analysis by dividing TAPSE based on its median value showed that patients with TAPSE ≤ 16 mm had 2.227 times higher probability for having longer hospital LOS. Although previous studies by Ghio et al [13] and Damy et al [15] have reported the prognostic value of RV dysfunction (as measured by TAPSE) with the adverse outcomes in HF patients, those studies were limited to patients with chronic HF and reduced LV function. Taken together, the findings of this study and other studies suggested that evaluation of RV function should be considered as a part of comprehensive evaluation in hospitalized ADHF and chronic HF patients, and when available, should be used to stratify those patients for prognostic and therapeutic purposes.

The present study strengthens the previous observations by demonstrating the feasibility of a noninvasive echocardiographic evaluation of RV function as well as its usefulness in the prognostic stratification of patients with ADHF. The superior prognostic strength of TAPSE with respect to the other echocardiographic parameters of RV systolic function is that a reduced TAPSE better reflects a severe impairment of RV performance, because the systolic shortening of the RV from base to apex provides information not only on the emptying of the RV but also on the driving force which acts on the systemic venous column [13].

In addition, our study found the prevalence of WRF was 27.8 % and it confirmed previous studies that reported the occurrence of WRF to be approximately 27–34 % [20, 32], but it was higher than other studies that reported the prevalence of 9–15 % [33–36]. We found that WRF occured during hospitalization associated significantly with longer LOS, as also shown in other previous studies [1, 9]. Wright et al [1] reported that the development of renal failure were independently associated with longer than average LOS. This is due to the fact that comorbidities such as renal dysfunction will delay patient’s response to treatment, prevent the use of certain treatments and require additional time to manage [3].

Finally, we found an interesting obervation in which the prevalence of malnutrition in our ADHF patients was higher (49 %) than the study reported by Aziz et al [10] (34 % in ADHF patients in United States) and Al Najjar et al [37] (23 % in CHF patients in United Kingdom). This high prevalence showed that malnutrition was one of the frequent comorbidities that occured in heart failure patients. Although the mechanism on how nutritional status affects the evolution of HF has not been sufficiently established, several studies has reported that malnutrition was associated with worse outcome in HF patients. Aziz et al [10] reported that lower NRI score was associated with longer hospital LOS, higher readmission and mortality rates in ADHF patients. Another study also demonstrated that NRI was an independent predictor of mortality in ambulatory patients with CHF [37]. Our study demonstrated that lower NRI scores on admission can provide valuable information about risks of extended hospitalization in ADHF patients. This result confirmed previously reported studies that malnutrition was significantly associated with increased LOS [10, 11, 38–40].

### Study limitations

Variables influencing hospital LOS in HF include a broad range of socio-cultural and economic factors outside the scope of this study. We did not assess other factors that were difficult to measure such as patients’ compliance, mobility, readiness for discharge, and factors related with healthcare environment. While a biomarker NT-proBNP was not analyzed in this study because our national health insurance did not cover the cost of its examination.

We excluded patients with severe tricuspid regurgitation because the accuracy of the RV systolic parameter has not been established in such patients [41–43]. We also could not provide systolic pulmonary artery pressure (sPAP) measurement due to lack of data on inferior vena cava dilatation and collapsibility in our subjects. Furthermore, we did not assess the predictive performance of other echocardiographic indices of RV function.

## Conclusion

This prospective cohort study demonstrated that RV dysfunction as assessed by TAPSE was an independent predictors of longer hospital LOS in ADHF patients. We also found high prevalence of malnutrition and worsening renal function in our study population that were significantly associated with longer LOS. Since TAPSE can be easily and reliably measured in most patients, it should therefore be considered as part of routine echocardiographic examination in hospitalized ADHF patients.

## Abbreviations

ADHF, acute decompensated heart failure; CI, confidence interval; HR, hazard ratio; LOS, length of stay; mPAP, mean pulmonary artery pressure; NRI, nutritional risk index; RV, right ventricle/ventricular; TAPSE, tricuspid annular plane systolic excursion; WRF, worsening renal function
